# The Cross-Communication of Cuproptosis and Regulated Cell Death in Human Pathophysiology

**DOI:** 10.7150/ijbs.84733

**Published:** 2024-01-01

**Authors:** Kuan-Hao Tsui, Jui-Hu Hsiao, Li-Te Lin, Yi-Ling Tsang, Ai-Ning Shao, Chen-Hsin Kuo, Renin Chang, Zhi-Hong Wen, Chia-Jung Li

**Affiliations:** 1 Department of Obstetrics and Gynaecology, Kaohsiung Veterans General Hospital, Kaohsiung, Taiwan.; 2 Institute of BioPharmaceutical sciences, National Sun Yat-sen University, Kaohsiung, Taiwan.; 3 Department of Obstetrics and Gynaecology, National Yang-Ming University School of Medicine, Taipei, Taiwan.; 4 Department of Medicine, Tri-Service General Hospital, National Defense Medical Center, Taipei 114, Taiwan.; 5 Department of Surgery, Kaohsiung Municipal Minsheng Hospital, Kaohsiung, Taiwan.; 6 Institute of Physiological Chemistry and Pathobiochemistry and Cells in Motion Interfaculty Centre (CiMIC), University of Münster, 48149 Münster, Germany.; 7 Institute of Clinical Medicine, National Cheng Kung University, Tainan 704, Taiwan.; 8 Genomics Research Center, Academia Sinica, Taipei 115, Taiwan.; 9 Department of Emergency Medicine, Kaohsiung Veterans General Hospital, Kaohsiung, Taiwan.; 10 Department of Marine Biotechnology and Resources, National Sun Yat-sen University, Kaohsiung, Taiwan.

**Keywords:** Copper, Cuproptosis, Regulatory cell death, Pathophysiology

## Abstract

Copper (Cu) plays a crucial and diverse function in biological systems, acting as a cofactor at numerous sites of enzymatic activity and participating in various physiological processes, including oxidative stress regulation, lipid metabolism, and energy metabolism. Similar to other micronutrients, the body regulates Cu levels to ensure homeostasis; any disruption in Cu homeostasis may result in various illnesses. Cuproptosis causes proteotoxic stress and ultimately results in cell death by the binding of Cu ions to lipid-acylated proteins during the tricarboxylic acid cycle of mitochondrial respiration. Cu is not only involved in regulatory cell death (RCD), but also in exogenous factors that induce cellular responses and toxic outcomes. Cu imbalances also affect the transmission of several RCD messages. Therefore, this article presents a thorough examination of the mechanisms involved in Cu-induced RCD as well as the role of Cu complexes in its pathophysiology.

## 1. Introduction

Copper (Cu) is an essential transition metal that serves as a cofactor for various enzymes and participates in crucial biological processes that maintain normal cellular metabolism and growth. Under typical physiological and chemical conditions, Cu^+^ is reduced to Cu^2+^ 1. Cu ions play a role in numerous biochemical reactions by donating or accepting electrons 2. These ions also act as cofactors or structural components of proteins or enzymes that regulate several physiological processes such as mitochondrial respiration, energy metabolism, and antioxidants 3, 4. Imbalanced Cu ion levels lead to oxidative stress and abnormal cellular autophagy, resulting in various Cu-or Cu ion-related diseases 5. Exposure to extraterrestrial sources causes an imbalance in the intracellular Cu metabolic balance, leading to cytotoxic and organism-damaging effects 6. Regulated Cell Death (RCD) is a universal process in living organisms essential for maintaining tissue homeostasis and responding to various stressors. Its primary function is to eliminate non-functional or potentially harmful cells, contributing to overall biological equilibrium. RCD is critical for cell homeostasis, tissue remodeling, and disease processes. Unlike accidental cell death (ACD), which lacks control, RCD relies on dedicated molecular machinery. It operates during both physiological conditions, such as embryonal development (referred to as "programmed" cell death), and pathological conditions like infection. These RCD forms differ in duration, morphology, and inflammatory consequences, with complex and interconnected molecular mechanisms 7. However, the mechanisms of cytotoxicity and cell death induced by excessive Cu exposure are not yet fully understood. This form of programmed cell death induced by Cu, known as cuproptosis 8, is distinct from other RCD pathways and relies on mitochondrial respiration. In this article, we summarize the latest research on Cu metabolism and its involvement in exogenous exposure-induced RCD, as well as its regulatory mechanisms 9, 10. We also provide an outlook for Cu-dependent RCD research to offer new insights into interventions for the toxic damage and diseases associated with Cu metabolic dysfunction.

## 2. Role of Cu in Regulating Cellular Processes

The presence of Cu and its stability in living organisms are determined by the delicate balance between the amount and distribution of its different ionic forms 11. Cu exists in two distinct ionic states: Cu^+^ (sub-Cu ions, reduced) and Cu^2+^ (Cu ions, oxidized), which are involved in regulating various cellular, physiological, and pathological functions 12. Cu^2+^ present in the extracellular milieu binds to and governs the interplay between cell membrane receptors and growth factors, whereas intracellular Cu^+^ primarily exists in the Cu^+^ state, acting as a structural modifier and/or oxidative reducer, either directly or by inhibiting phosphatases, thereby modulating the initiation state of growth factors at the membrane receptor level. Cu^+^ in the cytoplasm directly regulates kinase activity by modifying its structure or inhibiting phosphatases. In the nucleus, Cu^+^ regulates gene expression and subsequent protein synthesis by binding to transcription factors 13.

### 2.1. Processes Involved in the Uptake and Distribution of Cu

**Figure 1:**
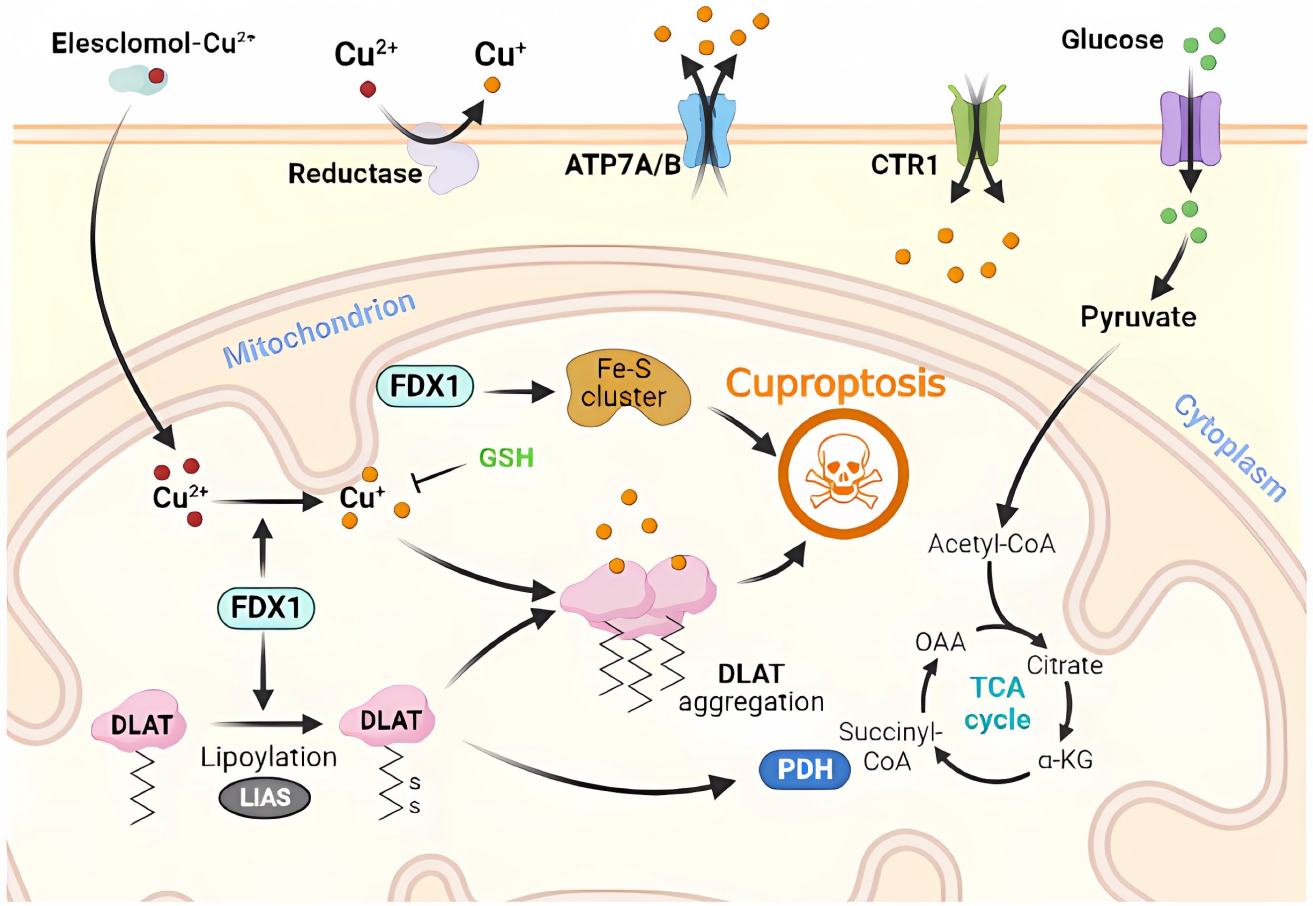
Molecular mechanisms of cuproptosis triggered by excess Cu. Elesclomol binds to Cu ions (Cu^2+^) in the extracellular environment and transports it to the intracellular compartment. Additionally, reductase enzymes convert Cu^2+^ to Cu^+^ to enable its entry into the cell. The accumulation of Cu inside the cell leads to Cu sagging, primarily through FDX1-mediated mitochondrial protein toxic stress. FDX1 reduces Cu^2+^ to Cu^+^ and promotes the lipidation and aggregation of enzymes that regulate the mitochondrial TCA cycle, especially DLAT. Conversely, FDX1 causes the destabilization of Fe-S cluster proteins. Apart from Cu ion carriers, Cu importers (e.g., SLC31A1) and exporters (e.g., ATP7B) affect intracellular Cu^+^ levels and modulate the sensitivity to Cu-induced apoptosis. The thiol-containing Cu chelator, GSH, blocks cuproptosis.

The distribution and stability of Cu in the body is determined by its absorption, transport, storage, and excretion within organisms and cells 14. It is mainly in the form of Cu^2+^ outside the cell and is reduced to Cu^+^ by reductive enzymes (e.g., the STEAP family) on the cell surface before absorption 15. Cu ions in the form of Cu^+^ are predominantly absorbed in the intestine, where the Cu transporter 1 (CTR1) trimer in the intestinal epithelial cell membrane plays a highly specific role in Cu^+^ uptake 16 (Figure 1). In the bloodstream, Cu binds to plasma proteins such as ceruloplasmin (CP), albumin, and transcopalbumin, which transport it to organs and tissues 13. Peripheral and liver tissue cells utilize Cu-transporting ATPase alpha (ATP7A) and b (ATP7B), respectively, for Cu uptake and efflux 17. ATP7A transports Cu to the portal vein, where it reaches the liver from the peripheral loop. Consequently, the liver acts as the primary trapper, distributor, and excretor of Cu; the latter subcellular distribution and targeting to various Cu proteins are regulated by the Golgi network and vesicles 16, 17. Cu contained within intestinal epithelial cells or hepatocytes contributes to the biosynthesis of Cu-zinc superoxide dismutase 1 (SOD1) in concert with Cu-specific partner proteins. SOD1 is a critical component of the antioxidant enzyme system that is indispensable to the maintenance of living organisms 18, 19. Cu in hepatocytes can be released into the bloodstream for distribution elsewhere or transported to the bile for excretion 20. ATP7A facilitates the passage of Cu across the blood-brain barrier into the brain, whereas ATP7B regulates the intracellular excretion of Cu. In the presence of excess Cu, Cu^+^ in the cytoplasm of hepatocytes is bound by the Cu partner protein antioxidant 1, which binds to the N-terminal metal-binding domain of ATP7B and is transferred to the bile duct membrane for the excretion of excess Cu from the body 21.

### 2.2. The Role of Cu in Mammalian Mitochondria

Mitochondrial function and Cu storage are closely linked 5, 17, and the Cu-binding protein, COX17, plays a critical role in the transfer of Cu^+^ from the cytoplasm to the mitochondrial intermembrane space. Cu is then passed to cytochrome oxidase-defective homologue 1 and next to the cytochrome C oxidase (CCO) II and I subunits, where it initiates enzyme activity in the respiratory chain. The exact mechanism of cytosolic Cu^+^ transfer to the mitochondrial compartment remains unknown, although a novel Cu ligand from the mouse liver may be involved 1, 6. Nevertheless, imbalance in Cu homeostasis, such as the excessive accumulation or faulty transportation of Cu, may have detrimental effect on cells when its intracellular concentration surpasses the threshold sustained by homeostatic mechanism 22, 23. Severe Cu deficiency may lead to impaired energy production caused by mitochondrial CCO dysfunction, whereas excessive intracellular Cu levels may contribute to the development of various diseases 24.

### 3. Molecular Mechanism of Cuproptosis

The intracellular accumulation of copper ions, often referred to as Cu, is a key contributor to the initiation of regulated cell death (RCD), a process known as "cuproptosis." Cuproptosis represents a specific form of RCD that is triggered when copper levels exceed a certain threshold within the cell. This excessive copper accumulation can occur due to various factors, including environmental exposure or disruptions in cellular copper homeostasis 25. Cuproptosis involves a rare post-translational modification of lysine, called protein lipidation, which results in the accumulation of lipoylated proteins that disrupt normal mitochondrial metabolism and induce RCD. In mammals, only four enzymatic complexes of lipoylated proteins have been identified, including those involved in the tricarboxylic acid cycle (PDH, alpha-ketoglutarate PDH, and KDH), branched-chain amino acid metabolism (BCKDH), and glycine metabolism (GCV) 26, 27. These enzymes are critical for maintaining normal mitochondrial metabolism and, when combined with Cu ions, lead to the accumulation of lipoylated proteins and mitochondrial metabolic dysfunction 20, 28.

Briefly, Cu^2+^ accumulates excessively in cells depending on mitochondrial respiration (Cu^2+^ is transported into the cell *via* Cu ion carriers), and it binds to thioredoxylated DLAT, inducing DLAT heterodimerization. Increased levels of insoluble DLAT lead to cytotoxicity and induce RCD 1, 29. FDX1 (a reductase, a direct target of elesclomol), as an upstream regulator of protein thioctylation modification, is involved in regulating the thioctylation of proteins, including DLAT, on the one hand 8, 23. In contrast, FDX1 reduces Cu^2+^ to the more toxic Cu^+^, leading to the inhibition of Fe-S cluster protein synthesis and the induction of RCD. Dysregulation of Cu homeostasis leads to RCD: Cu homeostasis is mainly dependent on three Cu transport proteins, SLC31A1, ATP7A/B, and SLC31A1, which are responsible for its uptake, and ATP7A and ATP7B, which are responsible for Cu export 30 **(Figure 1)**. The dysregulation of Cu homeostasis leads to cell death through a mechanism consistent with that of Cu ion carrier-induced RCD.

In the absence of extracellular Cu^2+^, elesclomol is not cytotoxic. When extracellular Cu^2+^ was chelated with a Cu chelator that was not permeable to the cell membrane, the uptake of Cu^2+^ and cytotoxicity of elesclomol was blocked 31. After the entry of Elesmol-Cu^2+^ complex into mitochondria, Cu^2+^ was reduced to Cu^+^ and reactive oxygen species were generated. After dissociation of the elesclomol-Cu^2+^ complex, elesclomol exits the cell to the extracellular compartment and forms a new elesclomol-Cu^2+^ complex, which transports Cu^2+^ from the extracellular compartment to the intracellular compartment, leading to the continuous accumulation of Cu in the mitochondria and eventually inducing apoptosis 32. It is worth noting that the mitochondrial selectivity exhibited by elesclomol is a unique feature of this compound that is not shared by other Cu chelators 32. Elesclomol has been shown to directly target FDX1, which encodes a reductase enzyme that reduces Cu^2+^ to the more toxic Cu^+^ 27. Zhang *et al* 33 have found that FDX1 is associated with ATP production and demonstrated that FDX1 is closely related to glucose metabolism, fatty acid oxidation, and amino acid metabolism. Similar to the roles played by FDX1, LIAS, LIPT1, and DLD are involved in fatty acid metabolism, whereas DLAT, PDHA1, PDHB, MTF1, GLS, and CDKN2A are associated with pyruvate dehydrogenase complex formation 34. Inhibition of FDX1, a crucial regulator of lipoylation-related proteins, prevents Cu-induced cell death 25. Cu toxicity is closely related to mitochondrial activity, which may be related to higher levels of lipoylated proteins in cells with vigorous mitochondrial metabolism and an active citric acid cycle 11.

These findings highlight the crucial role of FDX1 and fatty acid acylation in the regulation of Cu-induced RCD, a highly conserved post-translational lysine modification from bacteria to mammals 27, 33. Single-knockout experiments have shown that deleting FDX1 and LIAS leads to cellular resistance to Cu-induced RCD, and database analysis has confirmed a direct correlation between FDX1 and fatty acid acylation 26. Furthermore, the expression of iron-sulfur cluster proteins is reduced and their abnormal assembly during Cu-induced cell death has been reported 35. Iron-sulfur cluster proteins play important roles in various cellular processes such as electron transport, maintenance of genomic stability, and regulation of gene expression 12, 28. Higher levels of lipoylated proteins in cells with active tricarboxylic acid cycle and high mitochondrial metabolism may explain the close relationship between Cu toxicity and mitochondrial activity 15, 36. The mechanism underlying Cu-induced RCD involves the direct binding of Cu ions to lipidated proteins of the mitochondrial tricarboxylic acid cycle during respiration, which leads to the accumulation of lipidated proteins. This, in turn, results in the downregulation of the expression of iron-sulfur cluster proteins, causing proteotoxic stress, ultimately leading to RCD 25, 37.

## 4. The mechanism underlying Cu-induced RCD

Cu is involved in the activity of several metalloenzymes and plays a vital role in cellular mitochondrial respiration 22. Excess intracellular Cu is transported to the mitochondria *via* ion carriers and directly binds to lipid-acylated components of the mitochondrial respiratory tricarboxylic acid cycle 24. This process results in the aggregation of lipid-acylated proteins and loss of iron-sulfur cluster proteins, ultimately leading to differential RCD. In this article, we comprehensively examine the multifaceted regulatory cellular pathophysiology of Cu-induced apoptosis, necroptosis, pyroptosis, ferroptosis, and autophagy.

### 4.1. Molecular mechanism of Cu-induced apoptosis

**Figure 2:**
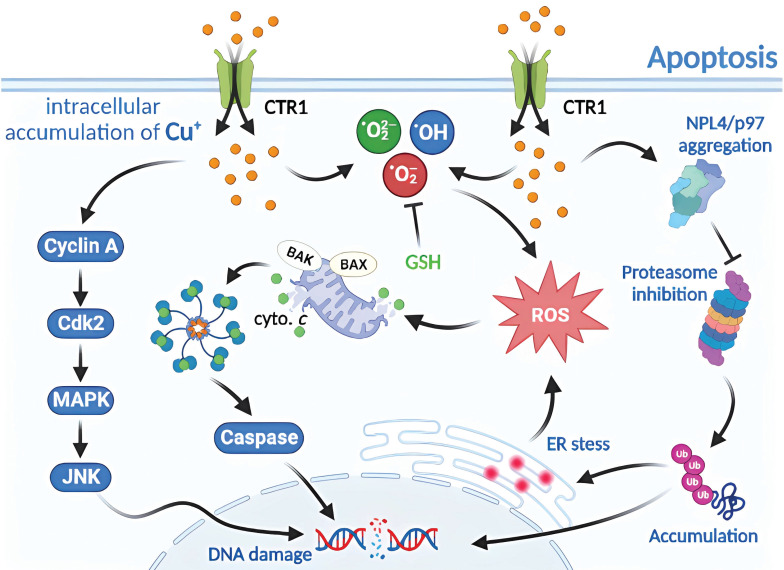
Molecular mechanisms of apoptosis triggered by excess Cu. Excess Cu is transported into cells *via* the Cu transporter, CTR1, resulting in a significant buildup of intracellular super radicals. This accumulation triggers cyclin A/Cdk2/MAPK/JNK activity and induces mitochondrial stress, causing the translocation of pro-apoptotic proteins (such as BAX and BAK) to the outer mitochondrial membrane. This event leads to the release of cytochrome *c* and the formation of apoptosome, which activates caspases and contributes to DNA damage. Cu also facilitates NPL4/p97 polymerization and inhibits the proteasome, leading to enhanced endoplasmic reticulum stress and ultimately resulting in apoptosis.

Apoptosis is a common form of programmed cell death or RCD in eukaryotic cells 7, 38. When the internal environment of a cell is disturbed by such as DNA replication errors or damage, endoplasmic reticulum stress, reactive oxygen species overload, or in response to external environmental stimuli, endogenous or exogenous apoptosis can be triggered to regulate cellular physiology and pathology 39, 40. Exposure of the body to large amounts of exogenous Cu substances and excessive uptake of Cu^2+^ increases the production of hydroxyl radicals and the level of intracellular reactive oxygen species, leading to enhanced lipid peroxidation, which in turn leads to oxidative stress and apoptosis 32. Furthermore, RCD induced by Cu ions can be amplified by CTR1 overexpression, and intracellular depletion of glutathione (GSH) and synaptic nuclear protein overexpression can aggravate Cu toxicity in dopaminergic cells by modulating protein degradation pathways 30, 41. Cu induces apoptosis *via* the endoplasmic reticulum unfolded protein response, endoplasmic reticulum stress, and oxidative stress. Wu *et al* 42 have found that the mRNA and protein levels of the CHOP, JNK, and Caspase-12 pathway molecules have been elevated in CuSO_4_-treated ICR mouse liver cells, activating the corresponding pathways to increase apoptosis and produce granular and vacuolar degenerative damage. Liu *et al*. 43 have further verified in CuSO_4_-treated mice that high doses of Cu^2+^ exposure induce oxidative stress through increased levels of reactive oxygen species and protein carbonyl compounds, and decreased GSH levels and mRNA and enzyme activities, such as SOD. These result in mitochondrial membrane potential depolarization, cytochrome *c* release, cleavage of caspase-9 and caspase-3, BAK and BAX levels increase and BCL-2 level decreases, inducing apoptosis **(Figure 2)**.

The ubiquitin-proteasome-mediated proteolytic system regulates the expression and activity of various proteins involved in cellular processes such as the cell cycle, proliferation, and apoptosis 44. Protein degradation by this proteolytic system involves two pathways: ubiquitination and degradation. Ubiquitination refers to the selective recognition of target proteins by the proteasome complex through ubiquitin tagging of target proteins for their degradation **(Figure 2)**. Currently, protease inhibitors, such as bortezomib, are widely used in the treatment of myeloma multiforme and have shown strong antitumor activity 45, 46. Disulfiram-Cu^2+^ complexes can block upstream signaling of the proteolytic system and impair the degradation of ubiquitinated proteins that are dependent on ubiquitination-dependent ATP synthase 47. Cu is more frequently recognized for its ability to trigger apoptosis through endogenous apoptosis-like stress damage associated with organelles, such as the mitochondria and endoplasmic reticulum. The modulation of organelle stress damage processes has the potential to disrupt Cu-induced apoptosis and influence regression 19, 48, 49.

### 4.2. Molecular mechanism of Cu-induced necroptosis

**Figure 3:**
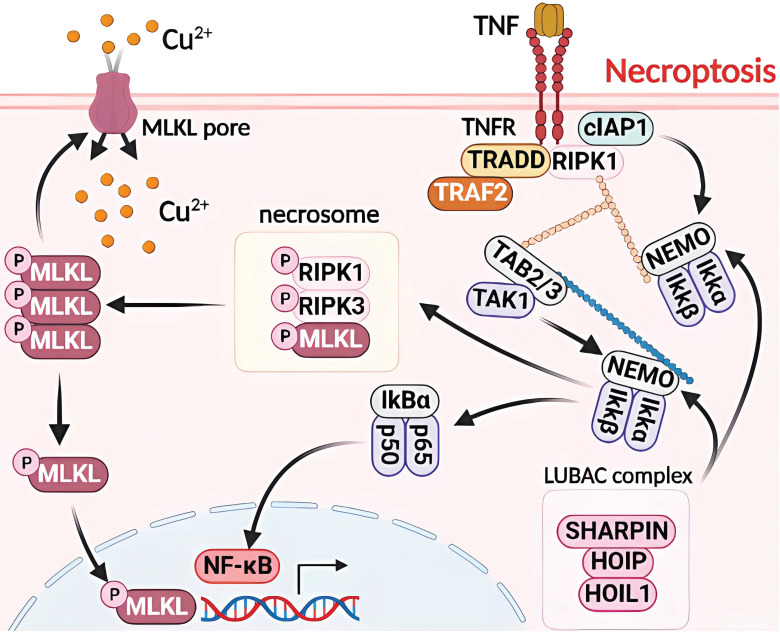
Molecular mechanisms of necroptosis triggered by excess Cu. Upon exposure to excessive Cu, cells activate the assembly of necroptosomes by allowing MLKL pore entry and upregulating MLKL levels. This not only provides components for MLKL pore assembly but also leads to MLKL translocation to the nucleus, thereby regulating NF-kB transcriptional activity. Cu overload also triggers downstream regulators of TNF/TNFR, including cIAP1/TRADD/RIPK1/TRAF2 complexes that regulate the protein levels of NEMO and TAB2/3, as well as IkBa activity, which in turn modulates NF-κB transcription. Furthermore, LUBAC is recruited to the activated TNF receptor complex by recognizing ubiquitin chains generated by other E3s, and it ligates linear chains to NEMO. The UBAN structural domain of NEMO in another IKK complex recognizes the linear chains bound to NEMO, resulting in the dimerization of the IKK complex, autophosphorylation of IKK2, and subsequent activation of NF-κB.

Necroptosis is a type of RCD with morphological similarities to necrosis, characterized by the loss of plasma membrane integrity, cytoplasmic translucency, increased cell volume, and organelle swelling 40, 50. Similar to apoptosis in mechanism, necroptosis is predominantly regulated by receptor-interacting protein kinase 1 (RIPK1), RIPK3, and mixed lineage kinase domain-like protein (MLKL), which collectively form a necrosome and play a crucial role as a signaling hub 38, 51. Cu-induced necroptosis and toxic damage through DNA damage, which depend on reactive oxygen species, have been observed. Cu ions are the most abundant metal ions in the nucleus. When cells are treated with CuCl_2_, Cu^2+^ enters the nucleus to bind to DNA and cause its breakage 52, 53. Necroptosis inhibitors, such as Nec-1 or z-VAD-FMK, have been shown to mitigate Cu^2+^-induced apoptosis. However, the toxicity of Cu^2+^ requires higher concentrations and longer duration of action than the cytotoxicity of Cd^2+^ to achieve toxicity levels similar to those of Cd^2+^ 38 **(Figure 3)**.

### 4.3. Molecular mechanism of Cu-induced pyroptosis

Pyroptosis is a form of RCD that is triggered by inflammatory vesicles that eliminate damaged cells and initiate inflammatory responses 10, 14. Various factors can induce pyroptosis, which is typically activated by injury or pathogen-associated molecular patterns that stimulate the NOD-like receptor family pyrin domain-containing protein 3 (NLRP3), bacterial infection that induces the CARD-containing NLR family protein 4 (NLRP3), activation of the NLR-family CARD-containing protein 4 (NLRC4) in response to bacterial infection, and the absence of the PYHIN family member in melanoma 2 (AIM2) in response to abnormal double-stranded DNA. Activation of these inflammasomes leads to the activation of caspase-1 and cleavage of pro-IL-1β and pro-IL-18 54.

**Figure 4:**
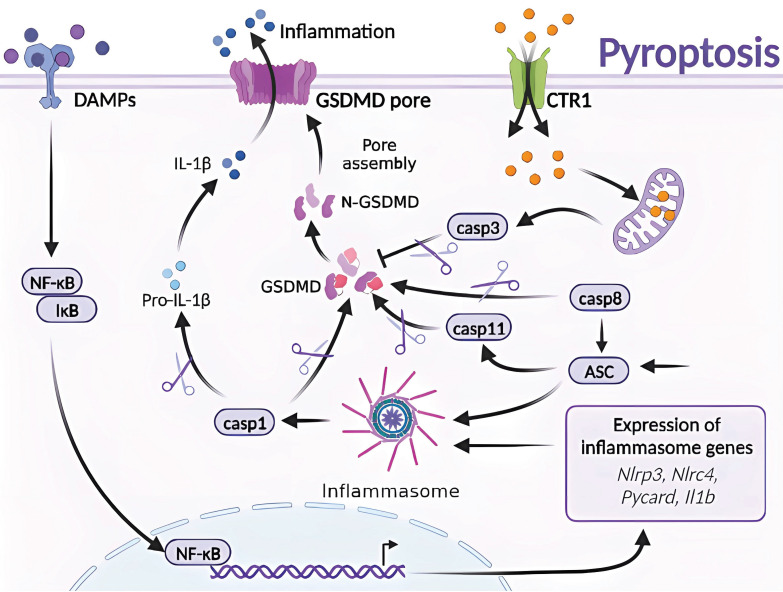
Molecular mechanisms of pyroptosis triggered by excess Cu. Excessive Cu influx through CTR1 leads to mitochondrial stress and activation of the caspase-3/GSDMD-dependent pyroptosis signaling pathway. GSDMD, downstream of caspase-3, forms an N-terminal domain that perforates the cell membrane, causing cell swelling, rupture, and release of inflammatory factors and DAMPs. Moreover, GSDMD is the effector protein of caspase-8/ASC/11, which undergoes cysteine cleavage to generate the N-terminal domain and promote pyroptosis. The GSDMD-dependent cell death pathway is mainly triggered by various aberrant signals, including the release of inflammatory factors that initiate a cascade of intracellular inflammatory responses (e.g., NF-κB).

Cu exposure induces NLRP3-dependent cellular pyroptosis, which mediates inflammatory responses and neurological toxicity 7. Deigendesch *et al*. have shown that Cu mediates macrophage pyroptosis and participates in the regulation of inflammatory response *via* the NLRP3 inflammatory vesicle-initiated pathway. In a mouse model of acute inflammation pretreated with the Cu chelator, TTM, serum caspase-1-dependent cellular factors were reduced, whereas caspase-1-independent cellular factors were not affected 55, 56. The impaired utilization and depletion of Cu in organisms may specifically inhibit caspase-1-dependent inflammasome activation and mediate systemic damage. Previous studies have also shown that the combined treatment of CuCl_2_ and lipopolysaccharide of primary microglia in non-mutant control mouse origin has revealed time-dependent increase in NLRP3, cleaved caspase-1, and ASC and IL-1β protein levels. CuCl_2_ exposure has been suggested to trigger NLRP3 activation-mediated inflammation and subsequent neurotoxicity in microglia 57. Hepatocytes are the major targets of Cu damage and toxicity. Excessive Cu exposure induces, caspase-1-dependent cellular pyroptosis, which mediates hepatocyte toxicity. Hepatocytes treated with CuSO_4_ and cocultured with N-acetylcysteine (NAC) have shown elevated mRNA levels of Cu^2+^-induced pyroptosis-related genes and caspase-1 protein expression. Treatment with the caspase-1 inhibitor Z-YVAD-FMK in combination with Cu^2+^ has resulted in a cell morphology closer to that of normal hepatocytes and attenuated Cu^2+^-induced an increase in lactate dehydrogenase, aspartate aminotransferase, and alanine aminotransferase activities, mitochondrial membrane potential, and reduced apoptosis. These results suggest a correlation and signaling pathway crosstalk between Cu exposure-induced apoptosis and pyroptosis 42, 57 **(Figure 4)**.

### 4.4. Molecular mechanism of Cu-induced ferroptosis

Ferroptosis depends on iron ions and is characterized by disturbance in iron homeostasis and the accumulation of reactive oxygen species in lipids 58. Two primary pathways that induce iron-dependent ferroptosis have been described 59. The first is the exogenous pathway, which involves the inhibition of cell membrane transport proteins such as cystine/glutamate transport proteins or the activation of iron transport proteins such as transferrin and lactotransferrin. The second is the endogenous pathway, which is initiated by the inhibition of intracellular antioxidant enzymes such as glutathione peroxidase 4 (GPX4) 10, 58, 60.

Previous studies have demonstrated that ferroptosis is modulated by various cellular metabolic processes, including homeostasis of redox and iron, mitochondrial activity, metabolism of amino acid, lipid, and glucose, and disease-related signaling pathways. Defense mechanisms and regulation of ferroptosis are supported by antioxidant systems located in different parts of the cell, such as the GPX4-GSH system in the cytoplasm and mitochondria 61.

**Figure 5:**
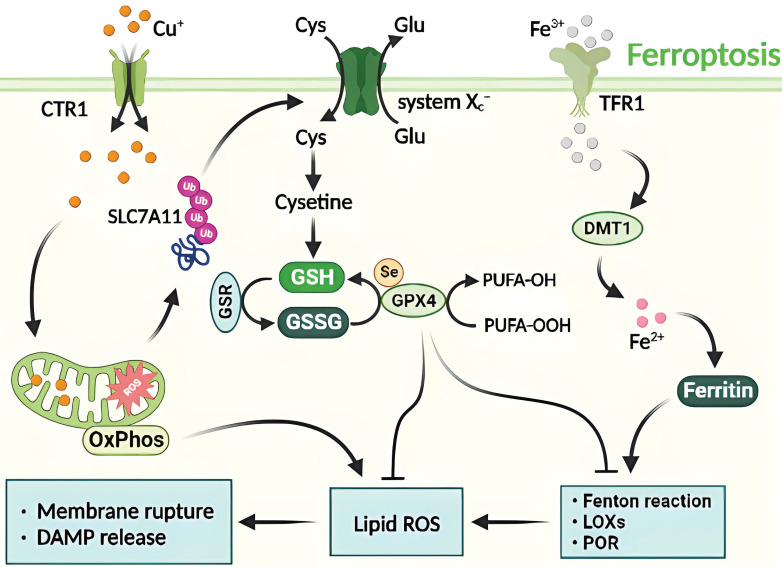
Molecular mechanisms of ferroptosis triggered by excess Cu. The transport of excess Cu into cells induces mitochondrial stress and activates the SLC7A11 signaling pathway, leading to ferroptosis. This process is triggered by the inhibition of systemic Xc- or GPX4 activity, ultimately resulting in cell death. The peroxidation of PUFA is believed to be a significant contributor to lipid ROS production during ferroptosis. Additionally, excess Cu-iron is essential for the execution of ferroptosis. It promotes the degradation of ATP7A11, leading to Cu retention in cells, ROS accumulation, and GPX4 activation. The latter is a key regulator of iron death that inhibits lipid ROS production by catalyzing the reduction of lipid hydroperoxides.

Despite not preventing elesclomol-induced RCD, the combined treatment of elesclomol and Cu in colon cancer cells results in the accumulation of reactive oxygen species and the degradation of solute carrier family 7 member 11 (SLC7A11), which is closely linked to ferroptosis. The administration of ferroptosis inhibitors has been shown to reduce elesclomol-induced RCD. It is hypothesized that Cu-induced RCD represents a form of Cu-dependent ferroptosis 25. This implies that elesclomol induces a unique type of Cu-dependent RCD that is impervious to apoptosis inhibitors and ferroptosis antagonists. An overabundance of Cu^+^/Cu^2+^ and Fe^2+^/Fe^3+^ can adversely affect cellular function by catalyzing the production of reactive hydroxyl radicals through the Fenton reaction, causing severe oxidative damage and RCD 37. Therefore, ferroptosis acts as a hub connecting metabolism, redox biology, and disease by facilitating RCD **(Figure 5)**.

### 4.5. Molecular mechanism of Cu-induced autophagy

Cellular autophagy, a type II programmed cell death process, can be classified into three types: microautophagy, molecular chaperone-mediated autophagy, and macroautophagy 62. Microautophagy involves direct wrapping and degradation of materials by the lysosomal membrane. Molecular chaperone-mediated autophagy involves the specific binding of target proteins to KFERQ motifs by the molecular chaperone HSP70 homologs, guiding them to the lysosome for degradation 63. Macroautophagy occurs in response to internal or external stimuli, in which a portion of the cytoplasm, damaged organelles, proteins, and other components are wrapped in a cytoplasmic inner membrane structure to form autophagosomes. Autophagosomes fuse with lysosomes to form autophagic lysosomes, and their contents are broken down by lysosomal hydrolases to meet the metabolic needs of cells and renew their organelles 60.

**Figure 6:**
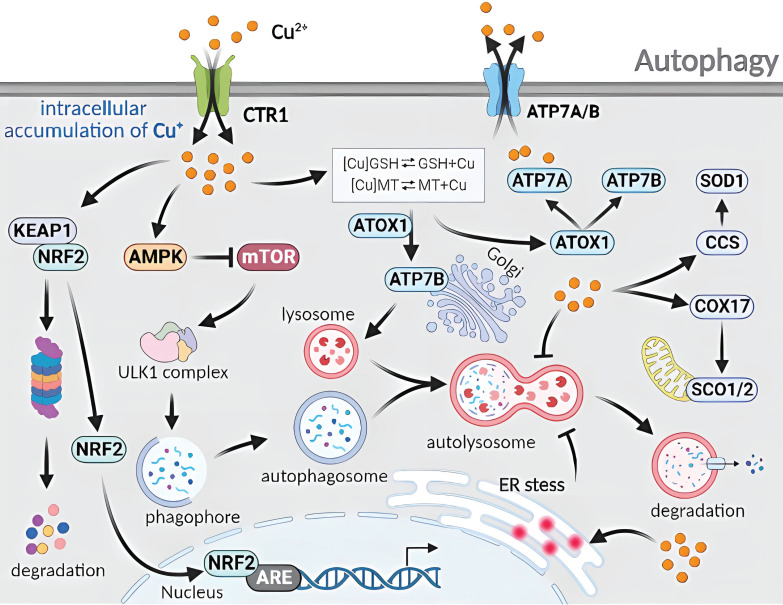
Molecular mechanisms of autophagy triggered by excess Cu. The cellular uptake of excess Cu through CTR1 regulates the autophagy mechanism. Cu increases the AMP/ATP ratio to inhibit mTOR and disrupts the binding between mTOR and ULK1 complexes. Cu also activates the CCS/SOD1 and COX17/SCO1/2 pathways, as well as the ATOX1/ATP7A/ATP7B pathways. The induction of endoplasmic reticulum stress by Cu disturbs the fusion phase of autophagy. Furthermore, Cu hinders the degradation phase of autophagy through a complex mechanism.

Cu-induced RCD occurs through the autophagy and mitochondrial autophagy pathways. When normal human lung bronchial epithelial cells are exposed to Cu^2+^, RNA microarray analysis has shown increased expression of heat shock response, ubiquitin-related, and autophagy-regulated genes. This implies that Cu^2+^ triggers proteasomal degradation, leading to the removal of misfolded and aggregated protein complexes from the cytoplasm *via* autophagy 19. In a study by Luo *et al*. 64, mouse mononuclear macrophages that have been treated with CuSO_4_ exhibit an increased level in mitochondrial reactive oxygen species which induce autophagy and autophagosome formation. Autophagy-related5 (Atg5) knockdown inhibits autophagy and enhances CuSO_4_-induced apoptosis. Yang *et al*. 65 have discovered that hepatocytes exposed to excess Cu display varying degrees of nuclear membrane disruption, chromatin condensation and fragmentation, and mitochondrial swelling and vacuolization, with an increase in the number of TUNEL-positive cells. This suggests that Cu induces mitochondrial damage and apoptosis. The increased number of TUNEL-positive cells indicate that Cu-induced apoptosis is associated with mitochondrial damage. Furthermore, mitochondrial autophagy triggered by the PINK1/Parkin pathway can reduce apoptotic damage. The regulation of mitochondrial damage through Cu imbalance triggers the activation of ATG-related proteins and promotes cell-initiated autophagy **(Figure 6)**.

## 5. Therapeutics Strategies for Targeting Cu-induced RCD

Therapeutic approaches to address Cu-induced regulated cell death, known as cuproptosis, encompass a wide array of interventions designed to prevent or alleviate the detrimental consequences of excessive copper accumulation inside cells. The objective of this field is to identify and create therapeutic methods that could offer potential treatments for disorders in which cuproptosis is a notable factor, ultimately aiming to enhance health outcomes and deter cell death resulting from copper overload.

### 5.1. Potential treatment against Cu-induced apoptosis

Previous research has shown that increased levels of Cu in the bloodstream and tumor tissues of cancer patients are strongly linked to the stage and progression of certain cancer types 19. Accumulation of Cu in cancerous tissues has become an attractive target for the development of anticancer drugs. Two main approaches have been tested to induce apoptosis by targeting Cu in preclinical and clinical settings. The first method involves the use of Cu chelators such as D-penicillamine, tetrathiomolybdate (TTM), and tretinoin to directly bind Cu and reduce its bioavailability. The second method uses Cu ion carriers such as disulfiram (DSF), docosahexaenoic acid, and thiosemicarbazone to increase intracellular Cu ion levels, generate reactive oxygen species, inhibit proteasomes, and induce apoptosis 32, 66.

Among these methods, one involves the binding of elesclomol to extracellular Cu, followed by the selective transportation of elesclomol-Cu^2+^ complexes into cells and their subsequent entry into mitochondria. In the mitochondria, Cu^2+^ is reduced to Cu^+^, which induces the generation of a significant number of reactive oxygen species, triggering apoptosis and resulting in an antitumor effect on diverse cancer cells 32. Additionally, it has been shown that DSF or metabolites conjugated with Cu are inhibitors of functional proteasome-related proteins in many cancers, leading to the accumulation of cytotoxic protein aggregates of polyubiquitinated proteins and important proteins such as IκB, p27, and c-Myc, resulting in cell cycle arrest and subsequent apoptosis 45. DSF-Cu complexes have been reported to possess the ability to inhibit NF-κB activity, induce apoptosis, sensitize cancer cells to anticancer drugs, and inhibit epithelial mesenchymal transition in hepatocellular carcinoma 67. Furthermore, researchers have improved the stability, metabolism, and plasma half-life of DSF by optimizing drug assembly and delivery strategies to exploit its ability to target Cu metabolic regulation and mediate its anticancer effect 68.

### 5.2. Potential treatment against Cu-induced necroptosis

Cu ions and compounds have the potential to induce necroptosis and may be utilized for tumor chemoprevention and targeted intervention 53. Plant compounds such as polyphenols are known to act on blood-separated lymphocytes, generating reactive oxygen species during the reduction of Cu^2+^ to Cu^+^ and its reoxidation, and binding chromatin, causing DNA breakage; in addition, the role of Cu^2+^ in DNA damage has been demonstrated using Cu chelators 69. Therefore, using compounds with selective cytotoxic properties for tumor cells (but not for non-tumor cells) with high Cu concentrations, it is possible to induce necroptosis and mediate tumor chemoprevention 70. The combination of CuS-NiS_2_ nanomaterials, which are used for clinical imaging diagnosis and have photothermal/photodynamic therapeutic properties, can induce the production of reactive oxygen species in human gastric cancer cells and initiate RIPK1/RIPK3/MLKL necrosome formation. Interference with MLKL decreases capping actin protein (CAPG) content, confirming that MLKL/CAPG-induced necroptosis significantly reduces tumor volume in mice 53. A Cu-mediated 18F-labeled positron emission tomography radiotracer, CNY-07, has been shown to target RIPK1 through the blood-brain barrier to characterize the occurrence of necroptosis in the brain and to perform *in vivo* imaging; in combination with RIPK1 inhibitors such as Nec-1 and DL747, it has been found to be more effective in the treatment of some brain diseases. Cu-related necroptosis has potential applications as a biomarker for disease diagnosis, drug evaluation, and molecular mechanism research 71.

### 5.3. Potential treatment against Cu-induced ferroptosis

Targeting the intracellular Cu-induced modulation of cellular ferroptosis can be applied to enhance the sensitivity of antitumor effects. Ferroptosis can be used as a target for cancer vulnerability 61. Cu stimulates ferroptosis through oxidative stress, suggesting that the design of new Cu complexes based on their structural and biological characteristics could make Cu complexes (elesclomol) and chelators potential anticancer agents 32. Gao *et al*. 72 have used elesclomol to increase Cu^2+^ levels in the mitochondria, decrease ATP7A expression, retain Cu^2+^, accumulate reactive oxygen species, promote SLC7A11 degradation, enhance oxidative stress, and induce iron chelation in colorectal cancer cells. DSF has great potential in human cancer therapy and can be used in combination with antitumor drugs to enhance their effects 73, 74. Sun *et al*. 75 have used synthetic FeCuS-lipid nanoparticles to induce ferroptosis in human gastric cancer cells, whereas Ren *et al*. 76 have found that a combination of DSF/Cu and sorafenib can inhibit tumor growth in an animal model of hepatocellular carcinoma. Yang *et al*. 77 have shown that regulating intracellular Cu-Fe homeostasis through the HIF1α/CP expression feedback loop can enhance HCC cell ferroptosis and radiosensitivity. Therefore, it is important to investigate the signaling pathways and molecular mechanisms involved in Cu-induced cellular ferroptosis to understand its role in toxic damage and tumor prevention.

### 5.4. Potential treatment against Cu-induced pyroptosis

The studies discussed here collectively encompass therapeutic approaches aimed at mitigating copper-induced pyroptosis, a regulated form of cell death associated with excessive copper accumulation. These strategies seek to ameliorate the adverse consequences of intracellular copper overload and offer potential treatments for conditions where copper-triggered apoptosis plays a pivotal role, ultimately leading to improved health outcomes. In the study by Liao *et al*., the focus was on understanding the impact of copper on hepatocyte pyroptosis and its intricate relationship with apoptosis. Their findings revealed that surplus copper induces pyroptosis through the generation of reactive oxygen species (ROS) within hepatocytes. The inhibition of caspase-1-dependent pyroptosis emerged as a potential therapeutic avenue for attenuating copper-induced apoptosis 78. Furthermore, the same group of researchers delved into the association between endoplasmic reticulum (ER) stress and pyroptosis in the context of copper-induced jejunal toxicity. Excessive dietary copper was found to induce both ER stress and pyroptosis in porcine jejunal epithelial cells. Interestingly, interventions targeting ER stress with specific inhibitors alleviated copper-induced pyroptosis, shedding light on a previously unexplored facet of copper toxicity 14. Deigendesch *et al*. embarked on an investigation into the role of intracellular copper in activating the NLRP3 inflammasome—a pivotal player in pyroptosis and inflammatory responses. Their results underscored the significance of intracellular copper homeostasis and hinted at the potential of copper chelators as a means to inhibit diseases reliant on NLRP3 inflammasome activation 55. Additionally, an associated study unveiled a pyroptosis inhibitor targeting the reactive cysteine within gasdermin D, the ultimate executor of pyroptosis downstream of inflammasome activation. These inhibitors exhibited the potential to suppress pyroptosis, IL-1β secretion, and inflammatory caspases, offering a versatile approach to alleviating inflammation and cell death associated with copper-induced pyroptosis 79. Collectively, these studies make substantial contributions to the development of therapeutic strategies aimed at addressing copper-induced pyroptosis, potentially opening up new avenues for treating diseases characterized by copper overload.

### 5.5. Potential treatment against Cu-induced autophagy

The underlying mechanisms of copper-induced lipid deposition, oxidative stress, and autophagy are discussed here, while potential therapeutic strategies are investigated to combat the deleterious consequences of copper overload. Xia *et al*., delved into the inhibitory effects of copper ions on ATG4B activity, subsequently leading to autophagy inhibition. The excessive presence of copper within cells induces oxidative stress and prompts the formation of insoluble Mallory bodies (MB). Their findings shed light on the potential therapeutic approach of curtailing copper-induced autophagy, thereby offering promise in addressing conditions like Wilson's disease (WD) 80. Zhong *et al*. explored how an excess of copper triggers alterations in oxidative stress levels, autophagic responses, and lipid metabolism. Their research highlights the pivotal role of oxidative stress-mediated Nrf2 activation in regulating copper-induced lipid accumulation. Autophagy is activated as a protective mechanism against the detrimental effects of copper-driven lipid buildup. These discoveries suggest that targeting Nrf2 could serve as a prospective therapeutic avenue for mitigating oxidative stress-related diseases such as obesity and non-alcoholic fatty liver disease (NAFLD)^81^.

The reciprocal regulation of NRF2 by autophagy and ubiquitin-proteasome mechanisms was examined as a means to modulate vascular endothelial injury induced by copper oxide nanoparticles (CuONPs). Exposure to CuONPs activates the NRF2 antioxidant pathway. This investigation uncovers an innovative regulatory mechanism, wherein autophagy not only promotes NRF2 activation but also, intriguingly, autophagy deficiency enhances proteasome-dependent NRF2 degradation. The comprehension of these intricate processes holds promise for the development of therapeutic strategies targeting CuONP-induced vascular injury and associated diseases 82. Furthermore, additional investigations have underscored the potential of quercetin in ameliorating copper-induced apoptosis and endoplasmic reticulum (ER) stress in SH-SY5Y cells by modulating autophagy. Copper exposure often triggers oxidative stress, apoptosis, and ER stress, all of which can lead to cellular damage. Quercetin, a polyphenol, emerges as a promising candidate for alleviating copper-induced toxicity by suppressing apoptosis and inducing autophagy. This study introduces a potential approach for mitigating the neurotoxicity associated with copper exposure 83. In summary, these studies collectively contribute to our comprehension of copper-induced autophagy and propose a range of therapeutic strategies to mitigate the adverse effects stemming from excessive copper accumulation within cells. These insights carry implications for the development of treatments for diseases linked to copper overload.

## 6. Conclusion and Future Perspectives

In conclusion, it is essential to maintain a dynamic balance of Cu ion levels in the body, as both excess and deficiency can lead to various diseases. Cu homeostasis is regulated by its absorption, transport, and excretion. Cu metabolism disorders have been associated with an increasing number of diseases, particularly in the field of oncology. Cuproptosis, a newly discovered type of RCD, is primarily associated with tricarboxylic acid cycle metabolic disorders. Cuproptosis is not entirely independent of other types of regulated cell death and may be closely related to them. Despite several studies providing meaningful insights into Cu-induced RCD, several questions remain unanswered. Different organs may require unique optimal Cu ion concentration, which is a crucial consideration in drug design and optimization. By answering these questions, we can gain a better understanding of the molecular mechanisms underlying human diseases related to cuproptosis, leading to improved investigations of Cu metabolism and its lethal mechanisms, as well as the screening for relevant drugs to treat Cu metabolic diseases.

## References

[1] Linder MC, Hazegh-Azam M (1996). Copper biochemistry and molecular biology. Am J Clin Nutr.

[2] Li Y, Trush MA (1993). DNA damage resulting from the oxidation of hydroquinone by copper: role for a Cu(II)/Cu(I) redox cycle and reactive oxygen generation. Carcinogenesis.

[3] Guo H, Wang Y, Cui H, Ouyang Y, Yang T, Liu C (2022). Copper Induces Spleen Damage Through Modulation of Oxidative Stress, Apoptosis, DNA Damage, and Inflammation. Biol Trace Elem Res.

[4] Jian Z, Guo H, Liu H, Cui H, Fang J, Zuo Z (2020). Oxidative stress, apoptosis and inflammatory responses involved in copper-induced pulmonary toxicity in mice. Aging (Albany NY).

[5] Cobine PA, Moore SA, Leary SC (2021). Getting out what you put in: Copper in mitochondria and its impacts on human disease. Biochim Biophys Acta Mol Cell Res.

[6] Kim BE, Nevitt T, Thiele DJ (2008). Mechanisms for copper acquisition, distribution and regulation. Nat Chem Biol.

[7] Nirmala JG, Lopus M (2020). Cell death mechanisms in eukaryotes. Cell Biol Toxicol.

[8] Chen L, Min J, Wang F (2022). Copper homeostasis and cuproptosis in health and disease. Signal Transduct Target Ther.

[9] Oliveri V (2022). Selective Targeting of Cancer Cells by Copper Ionophores: An Overview. Front Mol Biosci.

[10] Tong X, Tang R, Xiao M, Xu J, Wang W, Zhang B (2022). Targeting cell death pathways for cancer therapy: recent developments in necroptosis, pyroptosis, ferroptosis, and cuproptosis research. J Hematol Oncol.

[11] Chen X, Cai Q, Liang R, Zhang D, Liu X, Zhang M (2023). Copper homeostasis and copper-induced cell death in the pathogenesis of cardiovascular disease and therapeutic strategies. Cell Death Dis.

[12] Shi H, Jiang Y, Yang Y, Peng Y, Li C (2021). Copper metabolism in Saccharomyces cerevisiae: an update. Biometals.

[13] Grubman A, White AR (2014). Copper as a key regulator of cell signalling pathways. Expert Rev Mol Med.

[14] Liao J, Hu Z, Li Q, Li H, Chen W, Huo H (2022). Endoplasmic Reticulum Stress Contributes to Copper-Induced Pyroptosis via Regulating the IRE1alpha-XBP1 Pathway in Pig Jejunal Epithelial Cells. J Agric Food Chem.

[15] Niu YY, Zhang YY, Zhu Z, Zhang XQ, Liu X, Zhu SY (2020). Elevated intracellular copper contributes a unique role to kidney fibrosis by lysyl oxidase mediated matrix crosslinking. Cell Death Dis.

[16] Galler T, Lebrun V, Raibaut L, Faller P, Wezynfeld NE (2020). How trimerization of CTR1 N-terminal model peptides tunes Cu-binding and redox-chemistry. Chem Commun (Camb).

[17] Wang Y, Hodgkinson V, Zhu S, Weisman GA, Petris MJ (2011). Advances in the understanding of mammalian copper transporters. Adv Nutr.

[18] Li Y (2020). Copper homeostasis: Emerging target for cancer treatment. IUBMB Life.

[19] Ge EJ, Bush AI, Casini A, Cobine PA, Cross JR, DeNicola GM (2022). Connecting copper and cancer: from transition metal signalling to metalloplasia. Nat Rev Cancer.

[20] Chen J, Jiang Y, Shi H, Peng Y, Fan X, Li C (2020). The molecular mechanisms of copper metabolism and its roles in human diseases. Pflugers Arch.

[21] Narindrasorasak S, Zhang X, Roberts EA, Sarkar B (2004). Comparative analysis of metal binding characteristics of copper chaperone proteins, Atx1 and ATOX1. Bioinorg Chem Appl.

[22] Festa RA, Thiele DJ (2011). Copper: an essential metal in biology. Curr Biol.

[23] Arredondo M, Nunez MT (2005). Iron and copper metabolism. Mol Aspects Med.

[24] Scheiber I, Dringen R, Mercer JF (2013). Copper: effects of deficiency and overload. Met Ions Life Sci.

[25] Tsvetkov P, Coy S, Petrova B, Dreishpoon M, Verma A, Abdusamad M (2022). Copper induces cell death by targeting lipoylated TCA cycle proteins. Science.

[26] Rowland EA, Snowden CK, Cristea IM (2018). Protein lipoylation: an evolutionarily conserved metabolic regulator of health and disease. Curr Opin Chem Biol.

[27] Tsvetkov P, Detappe A, Cai K, Keys HR, Brune Z, Ying W (2019). Mitochondrial metabolism promotes adaptation to proteotoxic stress. Nat Chem Biol.

[28] Shanbhag VC, Gudekar N, Jasmer K, Papageorgiou C, Singh K, Petris MJ (2021). Copper metabolism as a unique vulnerability in cancer. Biochim Biophys Acta Mol Cell Res.

[29] Cox DW, Moore SD (2002). Copper transporting P-type ATPases and human disease. J Bioenerg Biomembr.

[30] Stern BR (2010). Essentiality and toxicity in copper health risk assessment: overview, update and regulatory considerations. J Toxicol Environ Health A.

[31] Guthrie LM, Soma S, Yuan S, Silva A, Zulkifli M, Snavely TC (2020). Elesclomol alleviates Menkes pathology and mortality by escorting Cu to cuproenzymes in mice. Science.

[32] Nagai M, Vo NH, Shin Ogawa L, Chimmanamada D, Inoue T, Chu J (2012). The oncology drug elesclomol selectively transports copper to the mitochondria to induce oxidative stress in cancer cells. Free Radic Biol Med.

[33] Zhang Z, Ma Y, Guo X, Du Y, Zhu Q, Wang X (2021). FDX1 can Impact the Prognosis and Mediate the Metabolism of Lung Adenocarcinoma. Front Pharmacol.

[34] Carreira-Barral I, Riopedre-Fernandez M, de Blas A, Mosquera J, Vazquez ME, Platas-Iglesias C (2020). Ditopic binuclear copper(II) complexes for DNA cleavage. J Inorg Biochem.

[35] Dreishpoon MB, Bick NR, Petrova B, Warui DM, Cameron A, Booker SJ (2023). FDX1 regulates cellular protein lipoylation through direct binding to LIAS. bioRxiv.

[36] Zhao G, Sun H, Zhang T, Liu JX (2020). Copper induce zebrafish retinal developmental defects via triggering stresses and apoptosis. Cell Commun Signal.

[37] Rakshit A, Khatua K, Shanbhag V, Comba P, Datta A (2018). Cu(2+) selective chelators relieve copper-induced oxidative stress *in vivo*. Chem Sci.

[38] Krumschnabel G, Ebner HL, Hess MW, Villunger A (2010). Apoptosis and necroptosis are induced in rainbow trout cell lines exposed to cadmium. Aquat Toxicol.

[39] Li CJ, Chu CY, Huang LH, Wang MH, Sheu LF, Yeh JI (2012). Synergistic anticancer activity of triptolide combined with cisplatin enhances apoptosis in gastric cancer *in vitro* and *in vivo*. Cancer Lett.

[40] Li CJ, Sun LY, Pang CY (2015). Synergistic protection of N-acetylcysteine and ascorbic acid 2-phosphate on human mesenchymal stem cells against mitoptosis, necroptosis and apoptosis. Sci Rep.

[41] Nose Y, Wood LK, Kim BE, Prohaska JR, Fry RS, Spears JW (2010). Ctr1 is an apical copper transporter in mammalian intestinal epithelial cells *in vivo* that is controlled at the level of protein stability. J Biol Chem.

[42] Wu H, Guo H, Liu H, Cui H, Fang J, Zuo Z (2020). Copper sulfate-induced endoplasmic reticulum stress promotes hepatic apoptosis by activating CHOP, JNK and caspase-12 signaling pathways. Ecotoxicol Environ Saf.

[43] Liu H, Guo H, Jian Z, Cui H, Fang J, Zuo Z (2020). Copper Induces Oxidative Stress and Apoptosis in the Mouse Liver. Oxid Med Cell Longev.

[44] Narayanan S, Cai CY, Assaraf YG, Guo HQ, Cui Q, Wei L (2020). Targeting the ubiquitin-proteasome pathway to overcome anti-cancer drug resistance. Drug Resist Updat.

[45] Kona FR, Buac D, A MB (2011). Disulfiram, and disulfiram derivatives as novel potential anticancer drugs targeting the ubiquitin-proteasome system in both preclinical and clinical studies. Curr Cancer Drug Targets.

[46] Cengiz Seval G, Beksac M (2018). The safety of bortezomib for the treatment of multiple myeloma. Expert Opin Drug Saf.

[47] Skrott Z, Mistrik M, Andersen KK, Friis S, Majera D, Gursky J (2017). Alcohol-abuse drug disulfiram targets cancer via p97 segregase adaptor NPL4. Nature.

[48] Filadi R, Pizzo P (2019). ER-mitochondria tethering and Ca(2+) crosstalk: The IP(3)R team takes the field. Cell Calcium.

[49] Saporito-Magrina CM, Musacco-Sebio RN, Andrieux G, Kook L, Orrego MT, Tuttolomondo MV (2018). Copper-induced cell death and the protective role of glutathione: the implication of impaired protein folding rather than oxidative stress. Metallomics.

[50] Santagostino SF, Assenmacher CA, Tarrant JC, Adedeji AO, Radaelli E (2021). Mechanisms of Regulated Cell Death: Current Perspectives. Vet Pathol.

[51] Tsui KH, Wang PH, Lin LT, Li CJ (2017). DHEA protects mitochondria against dual modes of apoptosis and necroptosis in human granulosa HO23 cells. Reproduction.

[52] Sepand MR, Aliomrani M, Hasani-Nourian Y, Khalhori MR, Farzaei MH, Sanadgol N (2020). Mechanisms and pathogenesis underlying environmental chemical-induced necroptosis. Environ Sci Pollut Res Int.

[53] Chen J, Zhang R, Tao C, Huang X, Chen Z, Li X (2020). CuS-NiS(2) nanomaterials for MRI guided phototherapy of gastric carcinoma via triggering mitochondria-mediated apoptosis and MLKL/CAPG-mediated necroptosis. Nanotoxicology.

[54] Kovacs SB, Miao EA (2017). Gasdermins: Effectors of Pyroptosis. Trends Cell Biol.

[55] Deigendesch N, Zychlinsky A, Meissner F (2018). Copper Regulates the Canonical NLRP3 Inflammasome. J Immunol.

[56] Tao X, Wan X, Wu D, Song E, Song Y (2021). A tandem activation of NLRP3 inflammasome induced by copper oxide nanoparticles and dissolved copper ion in J774A.1 macrophage. J Hazard Mater.

[57] Dong J, Wang X, Xu C, Gao M, Wang S, Zhang J (2021). Inhibiting NLRP3 inflammasome activation prevents copper-induced neuropathology in a murine model of Wilson's disease. Cell Death Dis.

[58] Jiang X, Stockwell BR, Conrad M (2021). Ferroptosis: mechanisms, biology and role in disease. Nat Rev Mol Cell Biol.

[59] Li CJ, Chang CH, Tsang YL, Fang SH, Chen SN, Chiang AJ (2022). Prognostic significance of ferroptosis pathway gene signature and correlation with macrophage infiltration in cervical squamous cell carcinoma. Int Immunopharmacol.

[60] Tang D, Kang R, Berghe TV, Vandenabeele P, Kroemer G (2019). The molecular machinery of regulated cell death. Cell Res.

[61] Lei G, Zhuang L, Gan B (2022). Targeting ferroptosis as a vulnerability in cancer. Nat Rev Cancer.

[62] Yim WW, Mizushima N (2020). Lysosome biology in autophagy. Cell Discov.

[63] Hansen M, Rubinsztein DC, Walker DW (2018). Autophagy as a promoter of longevity: insights from model organisms. Nat Rev Mol Cell Biol.

[64] Luo Q, Song Y, Kang J, Wu Y, Wu F, Li Y (2021). mtROS-mediated Akt/AMPK/mTOR pathway was involved in Copper-induced autophagy and it attenuates Copper-induced apoptosis in RAW264.7 mouse monocytes. Redox Biol.

[65] Yang F, Liao J, Yu W, Qiao N, Guo J, Han Q (2021). Exposure to copper induces mitochondria-mediated apoptosis by inhibiting mitophagy and the PINK1/parkin pathway in chicken (Gallus gallus) livers. J Hazard Mater.

[66] Tawari PE, Wang Z, Najlah M, Tsang CW, Kannappan V, Liu P (2015). The cytotoxic mechanisms of disulfiram and copper(ii) in cancer cells. Toxicol Res (Camb).

[67] Li Y, Wang LH, Zhang HT, Wang YT, Liu S, Zhou WL (2018). Disulfiram combined with copper inhibits metastasis and epithelial-mesenchymal transition in hepatocellular carcinoma through the NF-kappaB and TGF-beta pathways. J Cell Mol Med.

[68] McMahon A, Chen W, Li F (2020). Old wine in new bottles: Advanced drug delivery systems for disulfiram-based cancer therapy. J Control Release.

[69] Nanni V, Di Marco G, Sacchetti G, Canini A, Gismondi A (2020). Oregano Phytocomplex Induces Programmed Cell Death in Melanoma Lines via Mitochondria and DNA Damage. Foods.

[70] Chen W, Wang X, Zhao B, Zhang R, Xie Z, He Y (2019). CuS-MnS(2) nano-flowers for magnetic resonance imaging guided photothermal/photodynamic therapy of ovarian cancer through necroptosis. Nanoscale.

[71] Lan Y, Bai P, Liu Y, Afshar S, Striar R, Rattray AK (2021). Visualization of Receptor-Interacting Protein Kinase 1 (RIPK1) by Brain Imaging with Positron Emission Tomography. J Med Chem.

[72] Gao W, Huang Z, Duan J, Nice EC, Lin J, Huang C (2021). Elesclomol induces copper-dependent ferroptosis in colorectal cancer cells via degradation of ATP7A. Mol Oncol.

[73] Jiao Y, Hannafon BN, Ding WQ (2016). Disulfiram's Anticancer Activity: Evidence and Mechanisms. Anticancer Agents Med Chem.

[74] Li Y, Chen F, Chen J, Chan S, He Y, Liu W (2020). Disulfiram/Copper Induces Antitumor Activity against Both Nasopharyngeal Cancer Cells and Cancer-Associated Fibroblasts through ROS/MAPK and Ferroptosis Pathways. Cancers (Basel).

[75] Sun Y, An C, Wu L, Zeng W, Wang J, Wang Y (2021). Degradable FeCuS-Lipid Nanoparticles Confer Ultrasound-Activated CO Release and O(2)-Independent Radical Production for Synergistic Therapy. ACS Nano.

[76] Ren X, Li Y, Zhou Y, Hu W, Yang C, Jing Q (2021). Overcoming the compensatory elevation of NRF2 renders hepatocellular carcinoma cells more vulnerable to disulfiram/copper-induced ferroptosis. Redox Biol.

[77] Yang M, Wu X, Hu J, Wang Y, Wang Y, Zhang L (2022). COMMD10 inhibits HIF1alpha/CP loop to enhance ferroptosis and radiosensitivity by disrupting Cu-Fe balance in hepatocellular carcinoma. J Hepatol.

[78] Liao J, Yang F, Tang Z, Yu W, Han Q, Hu L (2019). Inhibition of Caspase-1-dependent pyroptosis attenuates copper-induced apoptosis in chicken hepatocytes. Ecotoxicol Environ Saf.

[79] Hu JJ, Liu X, Xia S, Zhang Z, Zhang Y, Zhao J (2020). FDA-approved disulfiram inhibits pyroptosis by blocking gasdermin D pore formation. Nat Immunol.

[80] Xia F, Fu Y, Xie H, Chen Y, Fang D, Zhang W (2022). Suppression of ATG4B by copper inhibits autophagy and involves in Mallory body formation. Redox Biol.

[81] Zhong CC, Zhao T, Hogstrand C, Chen F, Song CC, Luo Z (2022). Copper (Cu) induced changes of lipid metabolism through oxidative stress-mediated autophagy and Nrf2/PPARgamma pathways. J Nutr Biochem.

[82] Li N, Du H, Mao L, Xu G, Zhang M, Fan Y (2022). Reciprocal regulation of NRF2 by autophagy and ubiquitin-proteasome modulates vascular endothelial injury induced by copper oxide nanoparticles. J Nanobiotechnology.

[83] Chakraborty J, Pakrashi S, Sarbajna A, Dutta M, Bandyopadhyay J (2022). Quercetin Attenuates Copper-Induced Apoptotic Cell Death and Endoplasmic Reticulum Stress in SH-SY5Y Cells by Autophagic Modulation. Biol Trace Elem Res.

